# Family Dynamics, Socioeconomic Hardships, and Health Risk Behaviours of Bulgarian Adolescents during the COVID-19 Pandemic

**DOI:** 10.3390/children11081016

**Published:** 2024-08-20

**Authors:** Elitsa Dimitrova, Anna Alexandrova-Karamanova

**Affiliations:** Institute for Population and Human Studies—Bulgarian Academy of Sciences, 1000 Sofia, Bulgaria; annaalexandrova@yahoo.com

**Keywords:** cigarette smoking, e-cigarette use (vaping), alcohol use, drunkenness, cannabis use, COVID-19 pandemic, adolescents, socioeconomic hardships, family dynamics, Bulgaria, HBSC

## Abstract

Background/Objectives: This study aims to explore family dynamics and the economic hardships experienced by families during the COVID-19 pandemic and their associations with adolescents’ health risk behaviours (HRBs). Methods: Based on a representative study of adolescents aged 11–16 conducted in Bulgaria during the COVID-19 pandemic and HBSC data from the pre-pandemic period, logistic regression models were applied, assessing cigarette smoking, vaping, alcohol use, drunkenness, and cannabis use. The independent variables included demographics, Family Affluence Scale (FAS III), family structure, ease of communication with parents, and the authors’ developed questions on parents’ income and economic status change, family conflicts, and missing contact with extended family due to the pandemic. Results: Material status of the family showed increasing differentials in adolescents’ HRBs during the pandemic. Parental unemployment, income reduction, and temporary lay-offs were associated with a higher risk of substance use. Family conflicts, missing contact with extended family, and difficulties in communication with the mother were related to a higher risk of substance use. Communication with the father was significantly associated with alcohol use and drunkenness. Boys had lower odds of vaping and higher odds of alcohol use, drunkenness, and cannabis use. Higher age and minority status were associated with an increase in adolescents’ HRBs. Conclusions: This study highlights the need for special family-focused interventions in times of health and economic crises.

## 1. Introduction

Bulgarian adolescents had some of the highest rates of cigarette smoking, alcohol consumption, drunkenness, and cannabis use in the pre-pandemic period among 45 countries, participating in the 2017/2018 wave of the Health Behaviour in School-aged Children (HBSC) study [1]. Significant differences were uncovered by sociodemographic characteristics such as age, gender, family structure, and material status of the family, with young people from affluent families being more prone to health risk behaviours (HRBs) [2]. A systematic review of the factors associated with adolescents’ HRBs before the pandemic shows that peer influence, parental substance use, living in a single-parent family, easy access to harmful substances, parental styles (overprotection or loose parental control), and poor parental attachment were related to increased risk of alcohol use and drunkenness [3]. The factors significantly associated with cannabis use were peer influence, prior engagement in substance use (cigarette smoking and alcohol consumption), low parental monitoring, delinquent behaviours, and higher time spent in bars [3]. Depression, stress, poor academic achievements, parental and peer smoking, problems in family, conflicts with parents, and single parenting were identified as factors influencing cigarette smoking and vaping among young people. The use of nicotine products (regular cigarettes and heated tobacco products) was influenced also by restrictive policies and prohibitions of smoking in public places and easy access to nicotine products [3].

The COVID-19 pandemic reshaped everyday life, health, and wellbeing of adolescents and their families. In the case of Bulgaria, the pandemic was followed by an economic downturn related to lockdowns and restriction measures imposed during three major epidemic waves recorded in the country at the end of 2020 and 2021 and in early 2022 [4,5]. At that period, many families experienced economic hardships related to unemployment and the reduction in income and paid work of adult members [6,7]. The government implemented measures such as wage compensation schemes for companies and self-employed persons, aiming to support the economy in the period of imposed restrictions and interruption or reduction in work and business activities. Measures directly focused on families with (unemployed) adult members with dependent children were quite limited and included, for example, means-tested benefits for parents with children above 12 years who were at least 20 days in unpaid leave due to the pandemic and the provision of electronic devices for online schooling for children from ethnic minorities. Research shows that in families with increased financial strains during the pandemic, young people reported higher anxiety and depressive symptoms, decreased physical activity, unhealthy eating and higher engagement in substance use, prolonged screening time, and decreased academic performance [8,9,10,11,12]. In families with good relationships, the economic difficulties were less impactful, but in poor-functioning families experiencing financial difficulties, the lockdowns and stay-at-home measures restricted access to informal and formal support and exacerbated family conflicts, negatively affecting parents’ and children’s (mental) health [13,14,15,16,17,18].

Studies uncover different substance use patterns among adolescents during the COVID-19 pandemic. In certain contexts, the pandemic increased young people’s vulnerability and stress, making them more susceptible to substance -use, while in other contexts the imposed restrictions and stay-at-home measures were associated with a reduction in HRBs, explained manly with the limited access to substances, and decreased frequency of outdoor activities [19,20]. Substance use among adolescents depends also on the pre-pandemic history of use, changes in the contacts with peers and friends, and increased tensions and conflicts in the family during the pandemic [21,22,23,24,25].

The theoretical framework of the present study is based on Bowen’s Family Systems Theory [26,27]. According to this theory, the family system is an emotional entity featured by interconnectedness and mutual influence between family members. Changes in the emotional state of one family member influence the other family members. For example, parental anxiety and stress due to different life circumstances (e.g., unemployment, pressure at work, loss of income) might affect children’s emotional state and levels of anxiety. The theory takes into account not only the internal dynamics of family systems but also external system dynamics. Family is connected to a hierarchy of living systems, including individual, family, neighbourhood, community, society, and supranational system. The concept of the Societal Emotional Process developed by Bowen [26] posits that different societal processes affect the family. Periods of societal regression and progression affect family as an emotional unit and the relationships between family members. In periods of regression (e.g., in conditions of societal health crises caused by the COVID-19 pandemic and the following economic downturn), family dynamics might be negatively affected, causing emotional and behavioural problems in young people and problematic relationships with parents. Following this framework, the present study aims to explore the association between individual characteristics, family dynamics, and the socioeconomic hardships experienced by the families in Bulgaria during the COVID-19 pandemic and their associations with adolescents’ HRBs. We hypothesise that worsened family dynamics (family conflicts and limited contacts with close relatives due to the pandemic as well as difficulties in communication with parents) are associated with heightened risk of substance use (cigarette smoking, e-cigarette use [vaping], alcohol use, drunkenness, and cannabis use) among young people (H1). We expect also that the socioeconomic hardships (measured by the material status of the families, parental unemployment, temporary lay-offs, and decreases in income due to the pandemic) are associated with higher engagement of young people in substance use (H2). Our third hypothesis focuses on the interdependence between family dynamics (family conflicts) and the economic hardships experienced by the families during the pandemic. We expect that, in low-material-status families, frequent family conflicts will have a stronger negative effect on young people’s HRBs (H3).

## 2. Materials and Methods

### 2.1. Survey Design and Sampling Strategy

We use data from the Health Behaviour in School-aged Children (HBSC) study conducted in 2018 in Bulgaria. HBSC is a cross-national school-based study of adolescent health and wellbeing conducted in 51 countries at present. It aims to explore young people’s health and wellbeing, their determinants, and to inform youth policies [28]. The sample of HBSC-2018 is representative for the schoolchildren at ages 11, 13, and 15. After random selection of schools in all districts of the country, the survey was administered in class by an online questionnaire after obtaining parental consent. The national sample includes 4548 pupils (51.6% girls; mean age 13.5 (SD ± 1.65).

The study on adolescents’ lives, (mental) health, and wellbeing is a school-based survey that aims to explore the implications of the pandemic on adolescents’ (mental) health and wellbeing through young people’s self-reports. The implementation of the survey began at the end of school year 2021/2022 (June 2022) and continued in school year 2022/2023 after relaxation of the public health measures and reopening of the schools in Bulgaria from March 2022. The national representative sample includes 3345 students aged 11–16 years selected from a random school sample in the 28 districts of the country. The mode of data collection includes an online questionnaire completed by students in the schools after obtaining parental consent. The mean age of the students is 13.5 years (SD ± 1.74, girls—49.2%).

Both surveys have similar design and sampling strategy, with a slight difference in the covered age groups. This allows for group-level comparisons. More information about the design of the surveys, the sampling procedures, and the main variables can be found elsewhere [29].

### 2.2. Modelling Procedure and Variables Used in the Analysis

We apply logistic regression models in order to study the relationship between adolescents’ HRBs, family dynamics, and the socioeconomic hardships experienced by the families in Bulgaria during the COVID-19 pandemic. For comparison with the pre-pandemic period, we use logistic regression models applied to data from HBSC-2018 in order to outline the associations between selected sociodemographic and family-related variables and adolescents’ HRBs at that period. In addition, we explore the interaction between family affluence and family conflicts during the pandemic with an additional set of logistic regression models. The dependent variables in the models are cigarette smoking, e-cigarette use (vaping), alcohol use, excessive alcohol consumption (drunkenness), and cannabis use. We created a separate category for the missing values and controlled for them in the models.

Measures developed and validated within the HBSC study [28] were used to measure the prevalence of adolescents’ HRBs (dependent variables in the logistic regression models). Cigarette smoking is assessed by the question on how often a student smokes tobacco at present. The constructed variable includes the response option “I do not smoke” vs. combined response options, ranging from “Every day” to “Less than once a week”. The measure is identical in both surveys.

E-cigarette use (vaping) is assessed by the question of how many days (if any) an adolescent vaped (used e-cigarettes) in the past 30 days. The constructed variable includes the response option “Never” and “1–2 days” vs. the options, ranging from “3–5 days” to “30 days (or more)”. The measure of vaping is available only in the survey on adolescents’ lives, (mental) health, and wellbeing during the pandemic.

The composite variable, measuring alcohol consumption among young people, is assessed by the question on how often at present the student drinks anything alcoholic (beer, wine, spirits, alcopops, or any other alcoholic drink). The constructed variable includes the responses “Never”, “Rarely”, and “Every month” vs. the responses “Every day” and “Every week” for different alcoholic drinks. The measure is identical in both surveys.

Excessive alcohol consumption (drunkenness) is assessed by the question as to whether the adolescent had so much alcohol that they were really drunk in the last 30 days. The constructed variable includes the option “Never” or “1–2 days” vs. the responses, ranging from “3–5 days” to “30 days (or more)”. The measure is identical in both surveys.

Cannabis use is recorded only for 15-year-old students by the question on how many days (if any) the student smoked cannabis in the last 30 days. The constructed variable includes response option “Never” and “1–2 days” vs. the response options, ranging from “3–5 days” to “30 days (or more)”. The measure is identical in both surveys.

The independent variables, assessed at the individual level, include age recoded in two groups of 11–14- and 15–16-year-old students, gender and ethnicity in the study on adolescents’ lives, (mental) health, and wellbeing during the pandemic. In HBSC-2018, age is recoded in the following two groups: 11–13- and 15-year-old students. Ethnic belonging covers the main ethnic groups in the country (Bulgarian, Turkish, and Roma). The category “Other ethnic group” was recoded as missing due to the low number of cases. The question is available only in the study on adolescents’ lives, (mental) health, and wellbeing during the pandemic.

The family-related variables include family affluence assessed by the Family Affluence Scale, third revision (FAS III) [30]. The measure combines different material belongings in the family and distinguishes families with low, medium, and high material status. It is available in both surveys.

In HBSC-2018, parental employment status is recorded by the options “[Mother/father is…] In paid work”, “Mother/Father does not work”, and “I do not know (no mother/father).

In the study on adolescents’ lives, (mental) health, and wellbeing during the pandemic, the economic strains of the family are measured by the authors’ developed questions related to parental unemployment, temporary lay-offs, and reductions in income caused by the pandemic: “Due to the coronavirus (COVID-19) pandemic, have your mother/father lost their jobs/temporarily stopped working/was paid less?“ The response options are “Yes”, “No”, and “I do not know”.

Family dynamics during the pandemic uses the authors’ developed scale in measuring the relationships in the family. The following item is used in the present analysis: “In my family, we were more irritable and argued more often than before”. The 5-level response options range from “Never“ to “All the time”. The mean was used as a cut-off point, and two groups were created. The first group includes adolescents who never or sometimes experienced conflicts in the family vs. those who experienced family conflicts more frequently.

The experience of missing face-to-face contact with extended family members during the pandemic is part of the authors’ developed scale on social contacts and interactions with significant others during the pandemic. The following item is used in the present analysis: “During the pandemic, I missed face-to-face meetings and the time spent with my grandparents and other elderly or sick relatives”. The mean was used as a cut-off point, and two groups were created. The first group includes adolescents who never or sometimes missed contacts with close relatives vs. those who were missing these contacts more frequently.

The communication with parents is assessed separately for mother and father by questions with response options “Easy”, “Difficult”, and “I do not see mother/father (no mother/father)”. The questions are available in both surveys.

## 3. Results

The distributions in Table 1 show that, in the pre-pandemic period, the most prevalent HRB among adolescents was alcohol consumption (28.9%). Cigarette smoking and cannabis use were reported by 12.4% and 11.6% of the young people. Drunkenness was reported by 6.9%. In the pandemic period, the prevalence of HRBs increased (except for alcohol use and cannabis use). The prevalence of alcohol consumption decreased to 21.5% but drunkenness followed an upward trend, reaching 12.7%. Cigarette smoking increased to 19.2%, and the prevalence of vaping was 22.5%. Cannabis use was reported by 10.8% of the adolescents, remaining close to the prevalence in the pre-pandemic period.

In the pre-pandemic period, father’s unemployment/economic inactivity was reported by 2.7% of the adolescents, while mother’s unemployment/economic inactivity was somewhat higher, comprising 8.2% of the families. Of all adolescents, 3.9% reported that their father became unemployed due to the pandemic, and 4.1% reported the same for their mother. Temporary lay-offs due to the pandemic occurred more frequently in adolescents’ families. Father temporarily stopped working in 19.3% of the young people’s families, while mother’s temporary joblessness was 20.3%. A decrease in family income occurred in 16.5% of the families due to the father’s reduction in income caused by the pandemic and in 14.2% of the families due to the reduction in the mother’s income.

In both periods, students were found to experience more difficulties in the communication with father compared to the communication with mother. Difficulties in communication with mothers were reported by 12.2% of the young people in the pre-pandemic period. The percentage increased to 16.3% during the pandemic. Difficulties in communication with father were reported by 20.9% of the adolescents in the pre-pandemic period. During the pandemic, the same response was given by an almost equal share of the young people—21.8%.

### 3.1. Multivariate Analysis of Cigarette Smoking among Bulgarian Adolescents

The results from the models of cigarette smoking show that, before the pandemic, boys had significantly lower odds of cigarette smoking compared to girls (reference group) (Table 2, Model 1a). During the pandemic, gender differences ceased to be significant (Table 2, Models 1b and 1c). In both periods, higher age was significantly associated with higher odds of cigarette smoking. During the pandemic period, Roma adolescents were significantly more likely to smoke cigarettes compared to young people with Bulgarian ethnicity (reference group). In both periods, the risk of cigarette smoking significantly increased among students from single-parent families or living in foster care/with other relatives (reference group—students living with both parents). Family affluence was not in a significant relationship with cigarette smoking in the period before the pandemic. During the pandemic, adolescents from high-affluence families had significantly higher odds of cigarette smoking compared to students from medium-status families (reference group).

Before the pandemic, father’s unemployment/economic inactivity was associated with higher odds of cigarette smoking (reference category—fathers in paid work). The likelihood of smoking was significantly higher also among students who did not know if mother worked or had no mother (reference group—mother in paid work). Adolescents whose mother or father became unemployed due to the pandemic had a significantly higher risk of cigarette smoking (Table 2, Models 1b and 1c). The risk was higher also among students who did not know if father lost a job due to the pandemic (reference group—father did not become unemployed due to the pandemic). Students who did not know if their mother temporarily stopped working due to the pandemic and those whose fathers experienced a reduction in income had significantly lower odds of cigarette smoking.

Increased family conflicts during the pandemic are associated with higher odds of cigarette smoking. In both periods, young people who experienced difficulties in communication with mother had significantly higher odds of cigarette smoking (reference group—students who easily communicate with mother). The communication with father was not significant in the models on cigarette smoking.

In Model 2c, we include an interaction between family dynamics (conflicts in family during the pandemic) and family affluence. The results show that young people from low and high material status families who frequently experienced conflicts in family during the pandemic had significantly higher odds of cigarette smoking (reference group—students from medium-status families who did not or rarely experienced family conflicts during the pandemic).

### 3.2. Multivariate Analysis of E-Cigarette Use (Vaping) among Bulgarian Adolescents

The analysis of the factors related to e-cigarette use (vaping) is based on data from the period of the COVID-19 pandemic. The results presented in Table 3 Model 2a show that boys are significantly less likely to vape compared to girls (reference group). The increase in age is significantly associated with a higher risk of vaping. Young people from the Roma ethnic group have significantly higher odds of vaping compared to adolescents with Bulgarian ethnicity (reference group). Young people from single-parent families/living in foster care or with other relatives are significantly more likely to use e-cigarettes (reference group—students living with both parents). The odds of vaping are significantly higher among young people from well-off families compared to young people from medium-status families (reference group). Young people from families with low material status are significantly less likely to vape.

Students whose fathers became unemployed due to the pandemic and those who did not know about this had significantly higher risk of vaping (reference group—students whose fathers did not become unemployed due to the pandemic). Young people who did not know if their father temporarily stopped working due to the pandemic had significantly lower odds of vaping (reference group—students whose fathers temporarily stopped working due to the pandemic).

The risk of vaping is significantly higher among students who experienced frequent family conflicts during the pandemic. Young people who experienced difficulties in communication with mother or did not see her/have no mother have significantly higher odds of vaping (reference group—students who easily communicate with mother). The communication with fathers had no significant effect on the risk of vaping.

The interaction between family dynamics during the pandemic and family affluence presented in Model 2b shows that frequent family conflicts are associated with a higher risk of vaping among young people from less and high-affluent families (reference group—adolescents from medium-status families who did not or rarely experience family conflicts during the pandemic).

### 3.3. Multivariate Analysis of Alcohol Use among Bulgarian Adolescents

The results from the models on alcohol use presented in Table 4 show that, in both periods, before and during the pandemic, boys were more likely to report that they drunk alcohol compared to girls (reference group). The likelihood of alcohol consumption significantly increased with age. Adolescents from the Roma ethnic group had significantly higher odds of alcohol use compared to young people with Bulgarian ethnicity (reference group). In both periods, students from single-parent families and those living in foster care or with other relatives had significantly higher odds of alcohol use compared to young people living with a mother and father (reference group). In the pre-pandemic period, family affluence was not significantly associated with the risk of alcohol consumption (Model 3a). During the pandemic, students from low-status families had significantly lower odds of alcohol use, while those from affluent families had a significantly higher risk of alcohol use (reference group—adolescents from medium-status families).

In the pre-pandemic period, students who did not know if their mother works/have no mother were significantly more likely to drink alcohol compared to young people whose mothers were employed (reference group) (Model 3a). Students whose fathers were unemployed/economically inactive also had a significantly higher risk of alcohol use (reference group—adolescents whose fathers are employed).

Young people whose mothers became unemployed due to the pandemic had significantly higher odds of alcohol use (reference group—students whose mothers did not become unemployed due to the pandemic). Father’s unemployment caused by the pandemic is significantly associated with a higher risk of alcohol consumption among young people. Students whose mothers temporarily stopped working due to the pandemic showed significantly higher likelihood of alcohol use. Young people whose mothers were paid less due to the pandemic also had lower odds of alcohol use. The same relationship is found also among students who did not know if father experienced income reduction due to the pandemic.

Frequent family conflicts during the pandemic are related to significantly higher odds of alcohol use among young people. In both periods, before and during the pandemic, difficulties in communication with mother are associated with significantly higher likelihood of alcohol consumption (reference group—young people who easily communicate with mother). In the pre-pandemic period, young people who experienced difficulties in communication with father had significantly lower risk of alcohol use (reference group—students who easily communicate with father). In Models 3b and 3c about the pandemic period, the difference was not statistically significant.

In Model 3c, we test the interaction between conflicts in the family during the pandemic and the material status of the family. The results show that students from medium-status families who frequently experienced family conflicts were significantly less likely to use alcohol, while young people from affluent families who frequently experienced conflicts and tensions in the family had a significantly higher risk of alcohol use.

### 3.4. Multivariate Analysis of Excessive Alcohol Use (Drunkenness) among Bulgarian Adolescents

The results from the logistic regression models of excessive alcohol use (drunkenness) among Bulgarian students before and during the pandemic are presented in Table 5, Models 4a–c. In both periods, boys were found to have a significantly higher risk of drunkenness compared to girls (reference group). The association between age and excessive alcohol use is significant and positive. Roma adolescents have significantly higher odds of excessive alcohol use compared to young people with Bulgarian ethnicity (reference group). In the pre-pandemic period, family stricture was not significantly associated with the risk of drunkenness (Model 4a). During the pandemic, students from single-parent families and those living in foster care or with other relatives had significantly higher odds of excessive alcohol use.

In both periods, before and during the pandemic, students from high-affluent families were more likely to report excessive alcohol use (reference group—students from medium-status families). During the pandemic period, students from low-material-status families were significantly less likely to report excessive alcohol use (no statistical difference in the pre-pandemic period).

During the pre-pandemic, students who did not know if their mother works/have no mother had a significantly higher probability of excessive alcohol use (reference group—mother in paid work). Young people whose mothers became jobless due to the pandemic had significantly higher odds of drunkenness (reference group—students whose mothers did not become jobless due to the pandemic). The same relationship is found with respect to father’s unemployment caused by the pandemic. Students who did not know if father experienced income reduction due to the pandemic were less likely to report that they became drunk (reference group—students whose fathers did not experience income reduction due to the pandemic).

Family conflicts during the pandemic were significantly related to a higher risk of excessive alcohol consumption among young people. Adolescents who frequently missed contacts with close relatives also had a significantly higher likelihood of reporting excessive alcohol use. In both periods, young people who experienced difficulties in communication with their mothers were significantly more likely to report that they became drunk. In the case of communication with father, young people who did not see father/have no father were more likely to report excessive alcohol use (Models 4b and 4c).

The results from Model 4c, which includes interaction between family dynamics (conflicts in the family during the pandemic) and family affluence, show that students from affluent families who experienced frequent family conflicts during the pandemic had significantly higher odds of excessive alcohol use.

### 3.5. Multivariate Analysis of Cannabis Use among Bulgarian Adolescents

In Models 5a–c presented in Table 6, we explore the factors associated with cannabis use among 15-year-old students before and during the pandemic. In both periods, boys showed significantly higher odds of cannabis use compared to girls (reference group). Minority status (Turkish or Roma) is related to significantly higher risk of cannabis use (reference group—students with Bulgarian ethnicity). Family structure is seen to have a significant effect only in the period before the pandemic, showing that adolescents who lived in single-parent families or in foster care/with other relatives were more likely to smoke cannabis (reference group—students living with both parents). In the period before the pandemic, young people whose mothers did not work were significantly less likely to report that they used cannabis, while those in the category “I do not know/have no mother” had significantly higher risk (reference group—students whose mothers were in paid work).

Students reporting that mother became unemployed due to the pandemic or did not know about this had significantly higher risk of cannabis use (reference group—students whose mothers did not become unemployed due to the pandemic). Adolescents who did not know if father experienced income reduction due to the pandemic were less likely to report that they used cannabis (reference group—students whose fathers did not experience income reduction due to the pandemic).

Young people who frequently missed contact with close family members during the pandemic were significantly more likely to use cannabis. In both periods, before and during the pandemic, students who experienced difficulties in the communication with mother were significantly more likely to report that they used cannabis (reference group—students who easily communicate with mother). The variable about the communication with father was not statistically significant.

Model 5c shows that the interaction between family conflicts during the pandemic and family affluence is not significantly associated with cannabis use.

## 4. Discussion

This study aimed to explore family dynamics and socioeconomic hardships during the COVID-19 pandemic and their associations with substance use (cigarette smoking, vaping, alcohol use, drunkenness, and cannabis use) among young people in Bulgaria. Data from HBSC 2018 were used for comparisons with the pre-pandemic period. We selected factors at the persona and family level, including young people’s sociodemographic characteristics and family-related variables such as structure and material status of the family, parental unemployment, lay-offs, and reductions in income due to the pandemic, conflicts in the family, limited social interactions with close family members during the pandemic, and communication with parents. We explored also the interdependence between family dynamics (family conflicts during the pandemic) and family affluence.

The results from the multivariate analysis uncover strong differentials by gender, age, and ethnicity in substance use among young people in Bulgaria and changes in the effect of some of the sociodemographic dependencies during the pandemic. At that period, boys showed a higher risk of alcohol consumption, drunkenness, and cannabis use and a lower risk of vaping compared to girls. The difference between boys and girls was not statistically significant in cigarette smoking. In the pre-pandemic period, the risk of smoking was significantly higher among girls, and the gender difference in cannabis use was not significant. These results suggest changes in HRBs of boys and girls in Bulgaria that have occurred during the pandemic [2,31]. In a broader perspective, studies focused on substance use among young people reveal also diverging and inconsistent effects of gender in different contexts. Studies show that, rather, gender differences in coping with adverse experiences caused by the pandemic were differentiating factors in the disproportionate risk of substance use among boys and girls [8,32].

Similarly to the pre-pandemic period, substance use was prevalent among older adolescents in Bulgaria. This result shows that, despite the restrictions and the imposed social isolation, the older adolescents remained more susceptible to substance use [33,34]. In the pandemic period, substance use remained part of young people’s recreational and socialising activity and featured adolescents’ pathways to adulthood [35,36].

The present study uncovers a strong differentiating effect of ethnicity, with Roma adolescents having a higher likelihood of cigarette smoking, vaping, alcohol consumption, drunkenness, and cannabis use. Strong socioeconomic deprivation and social exclusion of Roma ethnic minority in Bulgaria was exacerbated during the pandemic as a consequence of the unfolding economic crisis, which increased the vulnerability of young people from this ethnic group, negatively affecting also young people’s health behaviours [37,38].

Family structure shows a consistent effect on adolescents’ HRBs in both periods, before and during the pandemic. Young people living with one of the parents/in foster care, or with other relatives were found to have a higher likelihood of cigarette smoking, vaping, alcohol consumption, drunkenness, and cannabis use. Studies show that increased difficulties experienced by single parents in combining different tasks during the pandemic had negative implications on parental and adolescents’ (mental) health and family relationships [39,40,41], emphasising also the need for family-focused help and support during the pandemic [42].

Bulgarian adolescents who frequently experienced family conflicts during the pandemic were found to have a higher risk of substance use (cigarette smoking, vaping, alcohol use, drunkenness). Young people who were frequently missing contact with close family members were more likely to use cannabis. Difficulties in communication with mother are associated with significantly higher risk of substance use among young people in both periods, before and during the pandemic. Difficulties in communication with father are in a significant relationship with the use of alcohol in the period before the pandemic and with drunkenness during the pandemic. The correlation between communication with mother and father may influence the significance of ease of communication with father. Monitoring and involvement of mothers in the daily routines and activities of adolescents may explain the significant effect of the communication with the mother on young people’s HRBs [43,44,45]. In countries with more traditional family culture, like Bulgaria, the father’s authority is built on perceptions of discipline, decision-making, material support, and provision for the family, while the mother is the person who is more involved in the daily routines of the family and care for children and adolescents [46,47,48]. These findings confirm our first hypothesis about the importance of family dynamics for young people’s HRBs during the pandemic.

Research shows that poverty and prolonged parental unemployment have a harmful effect on adolescent development, including also long-term negative consequences on health at later stages in life [49,50,51]. The results from the multivariate analysis reveal that, in the period before the pandemic, parental unemployment/economic inactivity was significantly associated with higher engagement of young people in cigarette smoking, vaping, alcohol consumption, drunkenness, and cannabis use. The economic hardships experienced by the families during the COVID-19 pandemic, related to parental joblessness, temporary lay-offs, and decreases in income caused by the pandemic, were associated with a heightened risk of cigarette smoking, vaping, alcohol consumption, drunkenness, and cannabis use among young people. The results reveal also increased differences in HRBs of students from different material status families during the pandemic. In the pre-pandemic period, the material status of the family had a significant effect on drunkenness, with students from affluent families having a higher risk for excessive alcohol use. During the period of the global health crisis, the risk of vaping, alcohol use, and drunkenness was significantly lower among students from less affluent families, and the effect was not significant in cigarette smoking and cannabis use. Heightened risk of substance use (cigarette smoking, vaping, alcohol use, and drunkenness) was observed among students from affluent families, and a non-significant effect was found for cannabis use. These results partly confirm our second hypothesis, revealing a disproportional effect of family affluence during the pandemic with an increased risk of substance use observed among young people from affluent families and a decrease in the risk among adolescents from materially disadvantaged families.

In order to test the interrelatedness between family dynamics (family conflicts) during the pandemic, family affluence, and adolescents’ HRBs, we applied additional models with interaction between family conflicts and the material status of the family during the pandemic. The results show that conflictual family relationships are associated with a higher likelihood of smoking, vaping, alcohol use, and drunkenness among young people from affluent families, while among students from low-material-status families the association is positive and significant only for cigarette smoking and vaping. These results partly confirm our third hypothesis. Conflictual family relationships among young people from less affluent families may strengthen the negative effect of economic hardships on young people’s HRBs. Among adolescents from high-material-status families, worsened family relationships and the availability of resources may increase young people’s engagement in substance use as a response to financial problems and conflicts in the family [52,53]. Studies show that economic difficulties leading to worsened parental mental health and increased tensions and conflicts in families during the pandemic were associated with heterogeneous responses in young people, including also maladaptive coping strategies of increased substance use [54,55,56,57,58]. In the case of Bulgarian adolescents, the economic hardships experienced by the families during the pandemic may affect young people’s behavioural health through increased anxiety and depression, provoking higher engagement in substance use [29,59].

## 5. Conclusions

The COVID-19 pandemic challenged the protective function of the family and reshaped young people’s lives in multiple directions. The health crisis created a specific environment of distress and strengthened socioeconomic difficulties for families, increasing the vulnerability of young people. The present study affirms that the economic hardships caused by parental unemployment, temporary lay-offs, and reductions in income are associated with a higher susceptibility to substance use as a maladaptive coping mechanism, especially among young people who experienced worsened family dynamics and increased conflictual relationships in the family during the COVID-19 pandemic.

### 5.1. Limitations of This Study

In the present analysis, we used cross-sectional surveys, which allows us to focus only on the associations between young people’s substance use, family dynamics (relationships in the family during the pandemic, missing contacts with extended family members, communication with parents), and the socioeconomic hardships experienced by the families during the pandemic. Both surveys include self-reported data that are subjected to response biases. The age groups of students in the surveys are slightly different, but the similarities in the design and in the measures used in the analyses allow for group-level comparisons. In HBSC-2018, parental unemployment and economic inactivity are combined in one category. In the study on adolescents’ (mental) health and wellbeing during the pandemic, parental unemployment, temporary lay-offs, and decreases in income caused by the pandemic are assessed for the whole period, which does not allow for a more differentiated look into the dynamics of parental economic status changes and their effect on adolescents’ HRBs. Ease of communication with parents is measured by a single question asked separately for mother and father. More complex measures are needed in order to outline more precisely the effect of openness and ease of communication with parents on adolescents’ HRBs.

### 5.2. Implications for Practice

The present analysis reveals that the socioeconomic hardships experienced by the families during the pandemic and worsened family relationships are related to higher engagement of young people in substance use (cigarette smoking, vaping, alcohol use, drunkenness, and cannabis use). These findings highlight the need for special interventions focused on different types of families in terms of material status, parental employment status, family dynamics, and parent-adolescent relationships. The results from this study can serve for the development of prevention programmes targeting the health behaviours of young people from families experiencing economic hardships and worsened family dynamics.

## Figures and Tables

**Table 1:** Descriptive statistics of the variables used in the analysis of Bulgarian adolescents’ HRBs before and during the COVID-19 pandemic.

Variables and Scales		2018		2021/2022	
	Response Options	N	Percent	N	Percent
	Not smoking	3985	87.6	2006	80.8
Cigarette smoking	Smoking	563	12.4	478	19.2
	Total	4548	100	2484	100
	Not vaping			1856	77.5
E-cigarette use (vaping)	Vaping			539	22.5
	Total			2395	100
	Not drinking	3159	71.1	1949	78.5
Alcohol consumption	Drinking	1283	28.9	535	21.5
	Total	4442	100	2484	100
	Did not get drunk	4235	93.1	2121	87.3
Excessive alcohol consumption (drunkenness)	Got drunk	313	6.9	309	12.7
	Total	4548	100	2430	100
	Did not use cannabis	1341	88.4	914	89.2
Cannabis use	Used cannabis	176	11.6	111	10.8
	Total	1517	100	1025	100
	Girl	2348	51.6	1637	49.6
Gender	Boy	2200	48.4	1667	50.5
	Total	4548	100	3304	100
	11–13 years	3031	66.6	2003	59.9
Age	HBSC—15 years/14–16 years	1517	33.4	1342	40.1
	Total	4548	100	3345	100
	Bulgarian			1976	83.6
Ethnicity	Turkish			147	6.2
	Roma			242	10.2
	Total			2365	100
	Two parents	3537	78.4	1635	74.5
Family structure	One parent/foster care/with Other relatives	974	21.6	559	25.5
	Total	4511	100	2194	100
	Low	1887	43.6	1272	57.6
Family affluence (Material status of the family)	Medium	760	17.6	382	17.3
	High	1680	38.8	555	25.1
	Total	4327	100	2209	100
	In paid work	3935	89.7		
Mother’s employment status	Mother does not work	358	8.2		
	I do not know/no mother	93	2.1		
	Total	4386	100		
	In paid work	4138	94.3		
Father’s employment status	Father does not work	119	2.7		
	I do not know/no father	130	3.0		
	Total	4387	100		
	No			2419	84.6
Mother lost job due to the pandemic	Yes			117	4.1
	I do not know/no mother			324	11.3
	Total			2860	100
	No			2467	84.8
Father lost job due to the pandemic	Yes			113	3.9
	I do not know/no father			329	11.3
	Total			2909	100
	No			1851	63.9
Mother temporarily stopped working due to the pandemic	Yes			589	20.3
	I do not know/no mother			459	15.8
	Total			2899	100
	No			1830	61.9
Father temporarily stopped working due to the pandemic	Yes			569	19.3
	I do not know/no father			556	18.8
	Total			2955	100
	No			1719	60.0
Mother paid less due to the pandemic	Yes			406	14.2
	I do not know/no mother			741	25.9
	Total			2866	100
	No			1615	55.4
Father paid less due to the pandemic	Yes			482	16.5
	I do not know/no father			820	28.1
	Total			2917	100
	No			1115	38.3
Family conflicts during the pandemic	Yes			1800	61.8
	Total			2915	100
	No			2636	89.9
Missing close relatives during the pandemic	Yes			296	10.1
	Total			2932	100
	Easy	3775	84.3	1846	81.0
Communication with mother	Difficult	546	12.2	372	16.3
	Do not see her/no mother	155	3.5	62	2.7
	Total	4476	100	2280	100
	Easy	3285	73.42	1639	71.5
Communication with father	Difficult	935	20.9	499	21.8
	Do not see him/no father	254	5.68	156	6.8
	Total	4474	100	2294	100

Notes: In the HBSC-2018 and the study on adolescents’ lives, (mental) health, and wellbeing during the pandemic, only 15-year-old students responded to the question about cannabis use. Age groups slightly differ in both surveys. The age groups in HBSC-2018 include 11–13- and 15-year-old adolescents. The study on adolescents’ lives, (mental) health, and wellbeing during the pandemic includes young people at ages 11–13 and 14–16 years. In HBSC-2018, parental employment status is measured in three categories: “In paid work”, “Mother/father does not work”, and “I do not know/no mother/father”. In the study on adolescents’ lives, (mental) health and wellbeing during the pandemic parents’ employment status is measured by joblessness, temporary lay-offs, and decreases in income caused by the pandemic.

**Table 2:** Logistic regression models of cigarette smoking among Bulgarian adolescents before and during the COVID-19 pandemic.

	Model 1a (Baseline)		Model 1b		Model 1c	
	OR		OR		OR	
Gender						
Girl (ref.)	1		1		1	
Boy	0.79 (0.65–0.95) **		1.01 (0.80–1.28)		1.01 (0.80–1.27)	
Age						
11–13 years (ref.)	1		1		1	
HBSC—15 years/14–16 years	5.68 (4.65–6.95) ***		3.91 (3.08–4.97) ***		3.96 (3.11–5.03) ***	
Ethnicity						
Bulgarian (ref.)			1		1	
Turkish			1.28 (0.80–2.06)		1.26 (0.78–2.02)	
Roma			2.53 (1.71–3.75) ***		2.70 (1.82–4.01) ***	
Family structure						
Two parents (ref.)	1		1		1	
One parent/foster care/with other relatives	1.80 (1.43–2.26) ***		1.99 (1.52–2.61) ***		1.95 (1.48–2.55) ***	
Family affluence (FAS III)						
Low	0.85 (0.62–1.15)		0.78 (0.54–1.11)			
Medium (ref.)	1		1			
High	0.98 (0.76–1.26)		1.35 (1.02–1.80) ***			
Mother’s employment status						
In paid work (ref.)	1					
Mother does not work	1.06 (0.74–1.54)					
I do not know/no mother	4.33 (2.50–7.51) ***					
Father’s employment status						
In paid work (ref.)	1					
Father does not work	2.64 (1.64–4.25) ***					
I do not know/no father	1.07 (0.62–1.84)					
Mother lost job due to the pandemic						
No (ref.)			1		1	
Yes			1.81 (1.02–3.21) **		1.80 (1.01–3.21) **	
I do not know			1.51 (0.86–2.64)		1.48 (0.84–2.58)	
Father lost job due to the pandemic						
No (ref.)			1		1	
Yes			2.90 (1.64–5.12) ***		2.90 (1.64–5.14) ***	
I do not know			2.44 (1.50–3.97) ***		2.49 (1.53–4.06) ***	
Mother temporarily stopped working due to the pandemic						
No (ref.)			1		1	
Yes			1.24 (0.88–1.74)		1.23 (0.88–1.73)	
I do not know			0.62 (0.37–1.06) *		0.63 (0.37–1.07) *	
Father temporarily stopped working due to the pandemic						
No (ref.)			1		1	
Yes			0.96 (0.67–1.37)		0.97 (0.68–1.38)	
I do not know			1.07 (0.69–1.67)		1.09 (0.70–1.70)	
Mother paid less due to the pandemic						
No (ref.)			1		1	
Yes			0.72 (0.47–1.10)		0.74 (0.48–1.12)	
I do not know			0.85 (0.56–1.27)		0.86 (0.57–1.29)	
Father paid less due to the pandemic						
No (ref.)			1		1	
Yes			0.85 (0.56–1.27)		0.88 (0.58–1.32)	
I do not know			0.63 (0.42–0.94) **		0.63 (0.42–0.95) **	
Family conflicts during the pandemic						
No (ref.)			1			
Yes			1.65 (1.26–2.15) ***			
Missing close relatives during the pandemic						
No (ref.)			1		1	
Yes			1.05 (0.72–1.51)		0.98 (0.68–1.42)	
Communication with mother						
Easy (ref.)	1		1		1	
Difficult	1.27 (0.97–1.65) *		2.70 (1.99–3.66) ***		2.72 (2.00–3.69) ***	
Do not see her/no mother	0.91 (0.56–1.48)		2.99 (1.57–5.69) **		2.82 (1.47–5.37) **	
Communication with father						
Easy (ref.)	1		1		1	
Difficult	1.09 (0.87–1.37)		1.07 (0.79–1.44)		1.07 (0.80–1.45)	
Do not see him/no father	0.96 (0.65–1.42)		1.01 (0.64–1.61)		1.04 (0.65–1.66)	
Family affluence (FAS III) * Family conflicts during the pandemic						
Low FAS * No family conflicts					1.52 (0.76–3.04)	
Medium FAS * No family conflicts (ref.)					1	
High FAS * No family conflicts					1.55 (0.91–2.64)	
Low FAS * Family conflicts					1.81 (1.24–2.63) **	
Medium FAS * Family conflicts					1.09 (0.66–1.78)	
High FAS * Family conflicts					2.41 (1.57–3.70) **	
Constant	0.05 (0.04–0.07) ***		0.04 (0.03–0.06) ***		0.04 (0.02–0.06) ***	
N	4548		2484		2484	
Log likelihood	−1474.49		−997.74		−997.14	
Pseudo R2	0.13		0.18		0.18	

Notes: (1) Missing values included as a separate category in the models. Results are not reported in this table. (2) OR—odds ratio. (3) Sig: ***: *p* < 0.001, **: *p* < 0.01, *: *p* < 0.05.

**Table 3:** Logistic regression models of e-cigarette use (vaping) among Bulgarian adolescents during the COVID-19 pandemic.

	Model 2a		Model 2b	
	OR		OR	
Gender				
Girl (ref.)	1		1	
Boy	0.78 (0.63–0.97) **		0.77 (0.62–0.96) **	
Age				
11–13 years (ref.)	1		1	
HBSC—15 years/14–16 years	2.59 (2.09–3.21) ***		2.60 (2.10–3.23) ***	
Ethnicity				
Bulgarian (ref.)	1		1	
Turkish	1.36 (0.87–2.12)		1.34 (0.86–2.09)	
Roma	2.28 (1.52–3.40) ***		2.29 (1.54–3.42) ***	
Family structure				
Two parents (ref.)	1		1	
One parent/foster care/with other relatives	1.96 (1.52–2.53) ***		1.93 (1.49–2.49) ***	
Family affluence (FAS III)				
Low	0.44 (0.30–0.65) ***			
Medium (ref.)	1			
High	1.25 (0.97–1.63) *			
Mother lost job due to the pandemic				
No (ref.)	1		1	
Yes	1.24 (0.69–2.20)		1.27 (0.71–2.25)	
I do not know	1.16 (0.69–1.95)		1.15 (0.68–1.93)	
Father lost job due to the pandemic				
No (ref.)	1		1	
Yes	2.06 (1.16–3.67) **		2.01 (1.13–3.57) **	
I do not know	1.81 (1.13–2.90) **		1.84 (1.15–2.94) **	
Mother temporarily stopped working due to the pandemic				
No (ref.)	1		1	
Yes	1.15 (0.84–1.58)		1.14 (0.83–1.56)	
I do not know	1.01 (0.63–1.61)		1.00 (0.63–1.61)	
Father temporarily stopped working due to the pandemic				
No (ref.)	1		1	
Yes	1.09 (0.79–1.50)		1.10 (0.80–1.51)	
I do not know	0.64 (0.42–0.98) **		0.65 (0.43–0.99) **	
Mother paid less due to the pandemic				
No (ref.)	1		1	
Yes	0.92 (0.63–1.36)		0.94 (0.64–1.38)	
I do not know	0.95 (0.66–1.38)		0.97 (0.67–1.40)	
Father paid less due to the pandemic				
No (ref.)	1		1	
Yes	0.80 (0.55–1.18)		0.81 (0.56–1.19)	
I do not know	0.75 (0.52–1.07)		0.74 (0.52–1.07)	
Family conflicts during the pandemic				
No (ref.)	1			
Yes	1.42 (1.13–1.80) **			
Missing close relatives during the pandemic				
No (ref.)	1		1	
Yes	0.92 (0.64–1.32)		0.87 (0.61–1.25)	
Communication with mother				
Easy (ref.)	1		1	
Difficult	2.20 (1.64–2.95) ***		2.21 (1.65–2.96) ***	
Do not see her/no mother	4.57 (2.50–8.37) ***		4.46 (2.43–8.18) ***	
Communication with father				
Easy (ref.)	1		1	
Difficult	1.19 (0.90–1.58)		1.20 (0.90–1.58)	
Do not see him/no father	0.97 (0.61–1.52)		0.98 (0.63–1.55)	
Family affluence (FAS III) * Family conflicts during the pandemic				
Low FAS * No family conflicts			0.67 (0.32–1.39)	
Medium FAS * No family conflicts (ref.)			1	
High FAS * No family conflicts			1.32 (0.84–2.07)	
Low FAS * Family conflicts			1.45 (1.05–2.01) **	
Medium FAS * Family conflicts			0.59 (0.36–0.95) **	
High FAS * Family conflicts			1.83 (1.26–2.67) **	
Constant	0.10 (0.07–0.13) ***		0.09 (0.07–0.13) ***	
N	2395		2395	
Log likelihood	−1119.13		1120.60	
Pseudo R2	0.12		0.12	

Notes: (1) The question of e-cigarette use is available only in the study on adolescents’ (mental) health and wellbeing during the pandemic. (2) Missing values included as a separate category in the models. Results are not reported in this table. (3) OR—odds ratio. (4) Sig: ***: *p* < 0.001, **: *p* < 0.01, *: *p* < 0.05.

**Table 4:** Logistic regression models of alcohol use among Bulgarian adolescents before and during the COVID-19 pandemic.

	Model 3a (Baseline)		Model 3b		Model 3c	
	OR		OR		OR	
Gender						
Girl (ref.)	1		1		1	
Boy	1.50 (1.31–1.72) ***		1.51 (1.22–1.87) ***		1.51 (1.22–1.87) ***	
Age						
11–13 years (ref.)	1		1		1	
HBSC—15 years/14–16 years	2.45 (2.13–2.82) ***		1.81 (1.47–2.24) ***		1.82 (1.47–2.25) ***	
Ethnicity						
Bulgarian (ref.)			1		1	
Turkish			1.04 (0.66–1.63)		1.02 (0.65–1.61)	
Roma			1.99 (1.37–2.90) ***		2.11 (1.44–3.09) ***	
Family structure						
Two parents (ref.)	1		1		1	
One parent/foster care/with other relatives	1.43 (1.21–1.70) ***		1.89 (1.47–2.44) ***		1.83 (1.42–2.37) ***	
Family affluence (FAS III)						
Low	0.96 (0.77–1.19)		0.63 (0.44–0.89) *			
Medium (ref.)	1		1			
High	1.08 (0.90–1.29)		1.27 (0.98–1.66) *			
Mother’s employment status						
In paid work (ref.)	1					
Mother does not work	0.93 (0.72–1.20)					
I do not know/no mother	2.33 (1.45–3.77) **					
Father’s employment status						
In paid work (ref.)	1					
Father does not work	1.60 (1.07–2.39) **					
I do not know/no father	1.14 (0.74–1.75)					
Mother lost job due to the pandemic						
No (ref.)			1		1	
Yes			2.38 (1.40–4.05) **		2.39 (1.40–4.07) **	
I do not know			1.18 (0.71–1.95)		1.18 (0.72–1.96)	
Father lost job due to the pandemic						
No (ref.)			1		1	
Yes			1.32 (0.76–2.29)		1.32 (0.76–2.30)	
I do not know			1.53 (0.95–2.44) *		1.56 (0.97–2.50) *	
Mother temporarily stopped working due to the pandemic						
No (ref.)			1		1	
Yes			1.35 (0.99–1.84) *		1.33 (0.98–1.82) *	
I do not know			1.11 (0.70–1.76)		1.09 (0.69–1.73)	
Father temporarily stopped working due to the pandemic						
No (ref.)			1		1	
Yes			1.19 (0.87–1.63)		1.19 (0.87–1.63)	
I do not know			0.77 (0.51–1.16)		0.78 (0.52–1.18)	
Mother paid less due to the pandemic						
No (ref.)			1		1	
Yes			0.62 (0.42–0.92) **		0.63 (0.42–0.93) **	
I do not know			1.29 (0.89–1.87)		1.29 (0.89–1.87)	
Father paid less due to the pandemic						
No (ref.)			1		1	
Yes			1.18 (0.82–1.70)		1.23 (0.85–1.77)	
I do not know			0.50 (0.34–0.72) ***		0.49 (0.34–0.72) ***	
Family conflicts during the pandemic						
No (ref.)			1			
Yes			1.31 (1.03–1.65) **			
Missing close relatives during the pandemic						
No (ref.)			1		1	
Yes			1.09 (0.77–1.54)		1.04 (0.74–1.47)	
Communication with mother						
Easy (ref.)	1		1		1	
Difficult	1.36 (1.11–1.66) **		2.09 (1.55–2.82) ***		2.09 (1.55–2.82) ***	
Do not see her/no mother	0.95 (0.65–1.38)		3.73 (2.04–6.83) ***		3.52 (1.91–6.48) ***	
Communication with father						
Easy (ref.)	1		1		1	
Difficult	0.83 (0.70–0.99) **		0.95 (0.71–1.27)		0.96 (0.72–1.28)	
Do not see him/no father	0.82 (0.60–1.11)		0.71 (0.44–1.15)		0.74 (0.46–1.19)	
Family affluence (FAS III) * Family conflicts during the pandemic						
Low FAS * No family conflicts					1.22 (0.66–2.25)	
Medium FAS * No family conflicts (ref.)					1	
High FAS * No family conflicts					1.06 (0.67–1.68)	
Low FAS * Family conflicts					1.31 (0.94–1.81)	
Medium FAS * Family conflicts					0.62 (0.38–0.99) **	
High FAS * Family conflicts					1.82 (1.25–2.66) **	
Constant	0.21 (0.18–0.24) ***		0.08 (0.06–0.11) ***		0.08 (0.06–0.12) ***	
N	4442		2484		2484	
Log likelihood	−2537.74		−1149.08		−1144.66	
Pseudo R2	0.05		0.11		0.12	

Notes: (1) Missing values included as a separate category in the models. Results are not reported in this table. (2) OR—odds ratio. (3) Sig: ***: *p* < 0.001, **: *p* < 0.01, *: *p* < 0.05.

**Table 5:** Logistic regression models of excessive alcohol use (drunkenness) among Bulgarian adolescents before and during the COVID-19 pandemic.

	Model 4a (Baseline)		Model 4b		Model 4c	
	OR		OR		OR	
Gender						
Girl (ref.)	1		1		1	
Boy	1.37 (1.08–1.74) **		1.85 (1.39–2.46) ***		1.84 (1.38–2.44) ***	
Age						
11–13 years (ref.)	1		1		1	
HBSC—15 years/14–16 years	2.68 (2.10–3.41) ***		2.18 (1.65–2.88) ***		2.18 (1.66–2.88) ***	
Ethnicity						
Bulgarian (ref.)			1		1	
Turkish			0.96 (0.52–1.77)		0.95 (0.52–1.74)	
Roma			2.05 (1.28–3.28) **		2.01 (1.26–3.22) **	
Family structure						
Two parents (ref.)	1		1		1	
One parent/foster care/with other relatives	1.23 (0.92–1.65)		2.88 (2.10–3.94) ***		2.81 (1.26–3.22) ***	
Family affluence (FAS III)						
Low	0.96 (0.65–1.42)		0.55 (0.34–0.88) **			
Medium (ref.)	1		1			
High	1.30 (0.96–1.75) *		1.63 (1.16–2.29) **			
Mother’s employment status						
In paid work (ref.)	1					
Mother does not work	0.64 (0.38–1.10)					
I do not know/no mother	2.95 (1.59–5.47) **					
Father’s employment status						
In paid work (ref.)	1					
Father does not work	1.68 (0.90–3.12)					
I do not know/no father	1.37 (0.73–2.54)					
Mother lost job due to the pandemic						
No (ref.)			1		1	
Yes			1.86 (0.97–3.56) *		1.90(1.00–3.6) **	
I do not know			1.46 (0.80–2.67)		1.41 (0.77–2.56)	
Father lost job due to the pandemic						
No (ref.)			1		1	
Yes			2.70 (1.43–5.07) **		2.66 (1.42–5.01) **	
I do not know			1.50 (0.87–2.59)		1.54 (0.89–2.66)	
Mother temporarily stopped working due to the pandemic						
No (ref.)			1		1	
Yes			1.00 (0.66–1.50)		0.99 (0.66–1.49)	
I do not know			0.84 (0.47–1.50)		0.86 (0.48–1.53)	
Father temporarily stopped working due to the pandemic						
No (ref.)			1		1	
Yes			1.32 (0.89–1.98)		1.31 (0.88–1.95)	
I do not know			0.83 (0.49–1.40)		0.82 (0.49–1.39)	
Mother paid less due to the pandemic						
No (ref.)			1		1	
Yes			0.72 (0.44–1.18)		0.74 (0.45–1.22)	
I do not know			1.00 (0.63–1.60)		1.02 (0.64–1.63)	
Father paid less due to the pandemic						
No (ref.)			1		1	
Yes			0.84 (0.52–1.35)		0.86 (0.53–1.38)	
I do not know			0.67 (0.42–1.08) *		0.67 (0.42–1.07) *	
Family conflicts during the pandemic						
No (ref.)			1			
Yes			1.52 (1.12–2.07) **			
Missing close relatives during the pandemic						
No (ref.)			1		1	
Yes			1.84 (1.23–2.76) **		1.74 (1.17–2.61) **	
Communication with mother						
Easy (ref.)	1		1		1	
Difficult	1.46 (1.06–2.01) **		2.50 (1.74–3.59) ***		2.52 (1.76–3.61) ***	
Do not see her/no mother	0.94 (0.51–1.72)		4.09 (2.11–7.90) ***		3.93 (2.03–7.63) ***	
Communication with father						
Easy (ref.)	1		1		1	
Difficult	0.90 (0.67–1.21)		0.97 (0.67–1.41)		0.98 (0.68–1.43)	
Do not see him/no father	1.28 (0.81–2.02)		1.52 (0.92–2.52) *		1.54 (0.93–2.55) *	
Family affluence (FAS III) * Family conflicts during the pandemic						
Low FAS * No family conflicts					0.65 (0.26–1.62)	
Medium FAS * No family conflicts (ref.)					1	
High FAS * No family conflicts					1.28 (0.69–2.38)	
Low FAS * Family conflicts					1.26 (0.81–1.96)	
Medium FAS * Family conflicts					0.70 (0.38–1.30)	
High FAS * Family conflicts					2.29 (1.41–3.72) **	
Constant	0.03 (0.03–0.04) ***		0.02(0.01–0.03) ***		0.02 (0.01–0.03) ***	
N	4548		2430		2430	
Log likelihood	−1076.34		−750.09		−753.68	
Pseudo R2	0.06		0.19		0.19	

Notes: (1) Missing values included as a separate category in the models. Results are not reported in this table. (2) OR—odds ratio. (3) Sig: ***: *p* < 0.001, **: *p* < 0.01, *: *p* < 0.05.

**Table 6:** Results from logistic regression models of cannabis use among Bulgarian adolescents during the COVID-19 pandemic.

	Model 5a (Baseline)		Model 5b		Model 5c	
	OR		OR		OR	
Gender						
Girl (ref.)	1		1		1	
Boy	1.35 (0.98–1.86) *		1.89 (1.11–3.21) **		1.82 (1.07–3.08) **	
Ethnicity						
Bulgarian (ref.)			1		1	
Turkish			3.87 (1.71–8.79) **		3.68 (1.63–8.33) **	
Roma			2.82 (1.22–6.50) **		2.94 (1.29–6.71) **	
Family structure						
Two parents (ref.)	1		1		1	
One parent/foster care/with other relatives	1.60 (1.05–2.41) **		1.52 (0.85–2.70)		1.48 (0.83–2.64)	
Family affluence (FAS III)						
Low	0.80 (0.45–1.40)		0.63 (0.29–1.40)			
Medium (ref.)	1		1			
High	1.26 (0.84–1.88)		1.48 (0.82–2.68)			
Mother’s employment status						
In paid work (ref.)	1					
Mother does not work	0.32 (0.11–0.92) **					
I do not know/no mother	3.05 (1.14–8.15) **					
Father’s employment status						
In paid work (ref.)	1					
Father does not work	1.31 (0.45–3.79)					
I do not know/no father	1.68 (0.62–4.56)					
Mother lost job due to the pandemic						
No (ref.)			1		1	
Yes			4.13 (1.57–10.87) **		4.32 (1.66–11.22) **	
I do not know			3.55 (1.25–10.07) **		3.73 (1.33–10.47) **	
Father lost job due to the pandemic						
No (ref.)			1		1	
Yes			5.31 (1.89–14.90) **		5.09 (1.81–14.30) **	
I do not know			1.87 (0.72–4.84)		1.86 (0.72–4.81)	
Mother temporarily stopped working due to the pandemic						
No (ref.)			1		1	
Yes			1.14 (0.54–2.40)		1.19 (0.57–2.50)	
I do not know			0.47 (0.15–1.53)		0.46 (0.14–1.48)	
Father temporarily stopped working due to the pandemic						
No (ref.)			1		1	
Yes			0.92 (0.44–1.90)		0.87 (0.42–1.81)	
I do not know			1.38 (0.51–3.72)		1.31 (0.49–3.55)	
Mother paid less due to the pandemic						
No (ref.)			1		1	
Yes			0.57 (0.23–1.45)		0.58 (0.23–1.48)	
I do not know			0.83 (0.34–1.99)		0.85 (0.35–2.07)	
Father paid less due to the pandemic						
No (ref.)			1		1	
Yes			1.06 (0.46–2.46)		1.05 (0.45–2.45)	
I do not know			0.39 (0.15–1.02) *		0.39 (0.15–1.01) *	
Family conflicts during the pandemic						
No (ref.)			1			
Yes			1.25 (0.71–2.21)			
Missing close relatives during the pandemic						
No (ref.)			1		1	
Yes			2.35 (1.21–4.57) **		2.26 (1.16–4.43) **	
Communication with mother						
Easy (ref.)	1		1		1	
Difficult	1.75 (1.16–2.64) **		4.32 (2.39–7.81) ***		4.31 (2.38–7.80) ***	
Do not see her/no mother	1.04 (0.47–2.31)		8.92 (2.90–27.42) ***		9.09 (2.94–28.12) ***	
Communication with father						
Easy (ref.)	1		1		1	
Difficult	0.90 (0.62–1.30)		0.62 (0.32–1.23)		0.62 (0.31–1.23)	
Do not see him/no father	0.56 (0.27–1.16)		0.84 (0.35–2.03)		0.84 (0.35–2.02)	
Family affluence (FAS III) * Family conflicts during the pandemic						
Low FAS * No family conflicts					0.54 (0.10–2.83)	
Medium FAS * No family conflicts (ref.)					1	
High FAS * No family conflicts					2.04 (0.70–5.93)	
Low FAS * Family conflicts					1.35 (0.60–3.02)	
Medium FAS * Family conflicts					0.86 (0.29–2.51)	
High FAS * Family conflicts					1.38 (0.54–3.49)	
Constant	0.09 (0.07–0.13) ***		0.02 (0.01–0.04) ***		0.02 (0.01–0.05) ***	
N	1517		1025		1025	
Log likelihood	−520.43		−256.97		−257.29	
Pseudo R2	0.04		0.27		0.27	

Notes: (1) Missing values included as a separate category in the models. Results are not reported in this table. (2) OR—odds ratio. (3) Sig: ***: *p* < 0.001, **: *p* < 0.01, *: *p* < 0.05.

## Data Availability

The data presented in this study are available upon request from the corresponding author. The data are not publicly available due to ethical restrictions (data contain sensitive personal information).
